# Changes in nutrient availability substantially alter bacteria and extracellular enzymatic activities in Antarctic soils

**DOI:** 10.1093/femsec/fiae071

**Published:** 2024-05-02

**Authors:** Girish R Nair, Bhaveni B Kooverjee, Storme de Scally, Don A Cowan, Thulani P Makhalanyane

**Affiliations:** Department of Microbiology, Faculty of Science, Stellenbosch University, Stellenbosch 7600, South Africa; Centre for Epidemic Response and Innovation, School for Data Science and Computational Thinking, Stellenbosch University, Stellenbosch 7600, South Africa; Department of Biochemistry, Genetics and Microbiology, University of Pretoria, Hatfield, Pretoria 0028, South Africa; Department of Biochemistry, Genetics and Microbiology, University of Pretoria, Hatfield, Pretoria 0028, South Africa; Centre for Microbial Ecology and Genomics, Department of Biochemistry, Genetics and Microbiology, University of Pretoria, Hatfield, Pretoria 0028, South Africa; Department of Microbiology, Faculty of Science, Stellenbosch University, Stellenbosch 7600, South Africa; Centre for Epidemic Response and Innovation, School for Data Science and Computational Thinking, Stellenbosch University, Stellenbosch 7600, South Africa

**Keywords:** antarctica, carbon, microcosm, nitrogen, soil, extracellular enzyme activities, climate change

## Abstract

In polar regions, global warming has accelerated the melting of glacial and buried ice, resulting in meltwater run-off and the mobilization of surface nutrients. Yet, the short-term effects of altered nutrient regimes on the diversity and function of soil microbiota in polyextreme environments such as Antarctica, remains poorly understood. We studied these effects by constructing soil microcosms simulating augmented carbon, nitrogen, and moisture. Addition of nitrogen significantly decreased the diversity of Antarctic soil microbial assemblages, compared with other treatments. Other treatments led to a shift in the relative abundances of these microbial assemblages although the distributional patterns were random. Only nitrogen treatment appeared to lead to distinct community structural patterns, with increases in abundance of *Proteobacteria* (*Gammaproteobateria*) and a decrease in *Verrucomicrobiota* (*Chlamydiae* and *Verrucomicrobiae*).The effects of extracellular enzyme activities and soil parameters on changes in microbial taxa were also significant following nitrogen addition. Structural equation modeling revealed that nutrient source and extracellular enzyme activities were positive predictors of microbial diversity. Our study highlights the effect of nitrogen addition on Antarctic soil microorganisms, supporting evidence of microbial resilience to nutrient increases. In contrast with studies suggesting that these communities may be resistant to change, Antarctic soil microbiota responded rapidly to augmented nutrient regimes.

## Teaser

Responses of Antarctic soil bacteria and their enzyme activity to augmented carbon and nitrogen

## Introduction

Soil microorganisms provide essential ecosystem services and are pivotal for the recycling of elemental carbon and nitrogen (Bardgett and Van Der Putten 2014, Bahram et al. 2018, Delgado-Baquerizo et al. 2018, Cavicchioli et al. 2019). Several studies have provided strong evidence regarding the global, regional, and local patterns of soil microorganisms (Serna‐Chavez et al. 2013, Delgado-Baquerizo et al. 2021, Shaffer et al. 2022). However, for several reasons including the logistics associated with studies in polar regions, comparatively less is known regarding Antarctic microbial communities (Makhalanyane et al. 2016). A recent global soil survey has provided strong evidence that colder high latitudinal are hotspots for soil nature conservation (Guerra et al. 2022). Yet, compared to more temperate soils, the effects of global warming induced climate change on the diversity and functional attributes of belowground soil microbial communities in the colder high latitudes remains largely unexplored.

Earlier studies report that nutrient rich soils harbor high microbial diversity (MD). These studies suggest that microbial communities play major roles in energy and nutrient flow (Miransari 2013, Tecon and Or 2017). There is some evidence that nutrient limitation profoundly impacts microbial food webs and soil formation (Krauze et al. 2021), biogeochemical cycling (Lysak et al. 2018), bioremediation (van Dorst et al. 2021), and ecological succession (Krauze et al. 2021). Restrictions on the availability of key soil nutrients, including organic carbon, may limit bacterial growth and directly affect microbial biomass and related enzymatic activities (Su et al. 2022). Similarly, nitrogen and water imbalances could also affect the abundance and respiration rates of soil microorganisms (Li et al. 2011). Previous studies show the importance of nitrogen and N-cycle on Antarctic soil bacterial community structure and related functions (Yergeau and Kowalchuk 2008, Berthrong et al. 2014, Lavergne et al. 2021). Reports from other polar habitats such as the Arctic have also seen shifts in diversity and functional attributes in permafrost soils as result of carbon fluctuations caused due to temperature changes (Monteux et al. 2018, Ricketts et al. 2020, Liu et al. 2021b). Nevertheless, the broader impacts on soil microorganisms remain unclear especially in soils from understudied cold environments. Given the fact that soil microorganisms in these environments underpin nutrient recycling, it is crucial to investigate the extent to which environmental stochasticity (e.g. nutrient availability fluctuations) impacts ecosystem services (Malard and Pearce 2018, Prather et al. 2019, Schmidt et al. 2022).

Pervasive melting of ice sheet in Antarctica due to climate change introduced by global warming has tremendously impacted the surface hydrology of Antarctica resulting in percolation and ablation zones. The increase in meltwater due to warming climate leads to runoff and mobilization of surface nutrients causing stoichiometric imbalances with impacts on microbial-derived ecosystem services (Bell et al. 2018, Soong et al. 2020). These imbalances may be especially significant in oligotrophic desert ecosystems, such as the Antarctic McMurdo Dry Valley (MDVs) soils where microbes dominate and prime biogeochemical cycling (Niederberger et al. 2019, Zoumplis et al. 2023). Developing an understanding of microbial responses to climate change has been a major focus of research over the past decade (Glassman et al. 2018, Malik et al. 2020, Wahid et al. 2020, Bardgett and Caruso 2020a). These studies suggest three types of responses to change including microbial resistance (remain in original state), resilience (change due to favorable adaptation), and functional redundancy (changes with unaltered ecosystem process rates) (Allison and Martiny 2008, Shade et al. 2011). These categories provide a valid framework for testing the impact of global change processes on nutrient cycling.

Evidence suggests that climate change-related changes in soil microbial activities may induce positive feedbacks (Frey et al. 2013, Nie et al. 2013), exacerbating the effects of change (Bardgett et al. 2008, Shakoor et al. 2020, Bardgett and Caruso 2020b, Fanin et al. 2022). For example, the effects of changes in soil microbial communities due to increases in nitrogen and phosphorous highlights significant changes in biogeochemical recycling (Rinnan et al. 2008, Campbell et al. 2010, Koyama et al. 2014, Ma et al. 2021). In addition to this, a recent study by Adamczyk et al. (2020) has demonstrated the impact of carbon addition on the abundances of Arctic soil microbial communities, using a combination of experimental manipulations and field studies. However, we lack broader insights regarding the effects of moisture and increased nitrogen and carbon inputs on the structure and function of microbial communities in oligotrophic Antarctic soils.

Here, we used soils from the MDVs to investigate the effects of nutrient supplementation. We predicted that the increased carbon, nitrogen, and soil moisture availability would substantially alter bacterial and archaeal communities, with direct impacts on the diversity and function. Using four treatments sets and one control, we simulated the effects of altered nutrient regimes and investigated the effects of nutrient augmentation on microbial communities over a period of 45 days via constructing soil microcosms in the laboratory. The treatments include supplementation with glucose (carbon source), ammonium chloride (nitrogen source), glycine (carbon and nitrogen source), and aerosolized filter sterilized water (moisture) to test the effects of higher carbon, nitrogen, and soil moisture, respectively. We used 16S rRNA gene amplicon sequencing to determine microbial community diversity dynamics in response to moisture and nutrient input. We also monitored extracellular enzymatic activities to evaluate microbial community-linked nutrient acquisition.

## Materials and methods

### Soil sampling and microcosm construction

Approximately 2 kg of bulk surface soils (0–5 cm) were collected during the austral summer of 2014, from a site near Spaulding Pond (77°39′S, 163°7′E), in the MDVs, Antarctica (Fig. S1) as described previously (Barnard et al.^2020^).The sampling site is situated in the MDV region of Eastern Antarctica, which is characterized by strong katabatic winds, minimal precipitation, and temperatures as low as −60°C during the austral winter (Sohm et al. 2020). The sampling was performed in a sterile manner from a 20 × 20 area by removing top 5 cm soil at an elevation of 68.7 cm and distance of ~8.35 m from the shoreline of Spaulding Pond in the Taylor Valley (Fig. S1). For the soil collection, all necessary permits were obtained from Antarctica New Zealand and the New Zealand Ministry of Foreign Affairs and Trade. These samples were placed into sterile Whirl-Pak bags (Nasco, WI, USA) and stored on ice, until transportation to the laboratory at the University of Pretoria in South Africa, where they were maintained at −80°C until further processing. The required amount of soil sample from several replicates were taken from −80ºC and samples were thawed slowly at −20ºC and then at 4ºC and then sieved to remove stones using 2 mm sterile (autoclaved) metal mesh, just before constructing microcosms. The methodology for microcosm construction was adopted from previously published work by our group (De Scally et al. 2016). Roughly each microcosm was constructed from 30 g of soil sample by randomly assigning the soil to four treatment groups each supplemented with carbon, nitrogen, carbon + nitrogen, and moisture in replicates of three. The untreated group was assigned as control for which no replicate was taken, and the nutrient and moisture sources were added only once at the start of the experiment and simulations were maintained for a period of 45 days with sample retrieval for analysis at intervals of 15, 30, and 45 days (Fig. S5). No sample was retrieved at day 0 for treated sets except for the controls (Table S1).

### Experimental manipulation, nutrient amendments, and chemical analysis

A randomized block design was used, and individual soil microcosms were placed in a Memmert ICP temperature-controlled incubator (Schwabach, Germany) at 15°C, under daylight conditions of ≥ 300 lx with forced air circulation and 70% humidity for a 45-day period. The relative humidity and temperature of the microcosms, and incubator, were monitored using iButton probes (Maxim Integrated, CA, USA), which were programmed to sample at 10-minute intervals. As opposed to untreated soils (control), treated soils were supplemented with aerosolized solutions of 0.85 M glucose (carbon source), 2.85 M ammonium chloride (NH4Cl; nitrogen source), 2 M glycine (carbon and nitrogen source), and filter-sterilized ultrapure water (0.15 g ml^−1^ w/v; moisture source). The treatments were applied at the beginning of the experiment excluding controls and not at regular intervals and samples were retrieved at intervals of day 15, 30, and 45, respectively (Fig. S5). We included 3 replicates per treatment × 4 treatment sets (carbon, nitrogen, carbon+nitrogen, and moisture) × 3 time points (15, 30, and 45) + 4 controls totaling 40 samples (Table S1). The supplements for nutrient sources (NSs) were selected based on their effectiveness in promoting growth and activity in soil microorganisms. Glucose addition can lead to carbon fixation in C-poor soils influencing soil bacterial diversity and function (Zhou et al. 2021, Karhu et al. 2022, Qi et al. 2022). Ammonium chloride is the considered as a steady and best nitrogen source for improving soil fertility and microbial growth outperforming other sources such as urea and ammonium nitrate (Wang et al. 2016, Shi et al. 2023). Glycine serves as a combined source of carbon and nitrogen, widely used in agricultural practices mitigating fertilizer requirement in soil and leads to improved utilization by soil microorganisms (Yang et al. 2016, Xue et al. 2022). At each sampling point, soils were aseptically removed from the microcosms, weighed, and stored in 50-ml Falcon tubes at −20°C until further analysis. The Coleman method (NT, 1984) was used to determine pH as previously described by Makhalanyane et al. (2013). The analysis of total organic carbon and nitrogen was performed by Bemlab laboratories (Somerset, South Africa) using a LECO Truspec® Elemental Determinator according to the instructions of the manufacturer.

### Molecular ecological analysis

#### DNA extraction and 16S rRNA gene sequencing

DNA was extracted from soil microcosms using the PowerSoil® DNA Isolation Kit as specified in the manufacturer's protocol (MO BIO Laboratories, CA, USA). For amplification, the V4–V5 region of the 16S rRNA gene was targeted, using primer pairs 515F (5′-GTGYCAGCMGCCGCGGRA-3′) and 909R (5′-CCCCGYCAATTCMTTTRAG-3′) (Tamaki et al. 2011). This amplification was followed by library preparation and sequencing at Molecular Research LP (MR DNA, Shallowater, TX, USA) using the Illumina MiSeq® platform as detailed previously (Caporaso et al. 2012). Demultiplexed amplicon sequence raw data obtained from the sequencing provider were processed using the default parameters in the *DADA2* pipeline (version 1.22) as described by Callahan et al. (2016). Quality control and error rate determination was performed, for each paired-end sequencing run, to account for run-specific errors. The quality control step involved trimming the low-quality sequences (Phred <20) from the reads using filter and trim parameter resulting reads with minimum read length of 190 bp. The resultant data were merged, and chimeric sequences were removed to obtain high quality sequences. The amplicon sequence variants (ASVs) table was generated from these sequences using *DADA2* algorithm that employs the error model for generating ASVs, which were analogous but more improved than OTU table differing only in single nucleotide over the sequenced region (Callahan et al. 2017). Further, taxonomic assignments were done using a native implementation of the naive Bayesian classifier method employed in *DADA2*. A sequence similarity of 97% against the Silva reference database was selected for comparisons to the Silva 138.1 prokaryotic SSU taxonomic training dataset. Finally, the data were filtered to remove mitochondrial and chloroplast derived sequences and singletons. These data were then rarefied to 836 (lowest library size) reads per sample to account for library size differences for downstream analysis. The Illumina MiSeq sequencing data are available on the NCBI-SRA under the BioProject accession PRJNA827358.

#### Extracellular enzyme assays

The effects of nutrient addition on soil microbiome function were determined by measuring extracellular enzymatic activities implicated in carbon, nitrogen, and phosphorus acquisition assays. Assays were performed as detailed by RL Sinsabaugh, CL Lauber, MN Weintraub, B Ahmed, SD Allison, C Crenshaw, AR Contosta, D Cusack, S Frey, and ME Gallo et al. (Sinsabaugh et al. 2008), with appropriate nonenzymatic controls. The dry mass of each soil sample was determined, after overnight incubation at 60°C and specific enzyme activities were calculated in units of nmol h^−1^g^−1^ dry mass and nmol h^−1^g^−1^ soil organic matter. Briefly, 5 g of soil was suspended in 100 ml 0.1 M Tris buffer, pH 8.6 (for samples with a pH greater than 8) or 0.1 M sodium acetate buffer, pH 5.5 (for samples with pH below 8). The resultant slurry was homogenized, and 200 μl was aliquoted into flat bottom 96-well microplates (Greiner, Frickenhausen, Germany; Corning Incorporated, New York, USA). In total, 50 μl of each substrate was added per well and four replicate wells were used per sample. For carbon acquisition, the activity of hydrolytic enzyme ß-1,4-glucosidase (BG) was measured by adding substrate 4-methylumbelliferyl-ß-d-glucosidase, again substrate 4-methylumbelliferyl- ß-d-xylosidase was added to detect ß-1,4-xylosidase (BX) enzyme. The activity of oxidative enzymes phenol oxidase (PO) and phenol peroxidase (PP) was measured by adding l-3,4-dihydroxyphenylalanine (l-DOPA) as substrate along with H_2_O_2_ for peroxidase activity. For nitrogen acquisition, substrates 4-methylumbelliferyl -*N*-acetyl-ß-glucosaminide l-leucine-7-amido-4-methylcoumarin were added to acquire the activity of ß-*N*-acetylglucosaminidase (NAG) and leucyl aminopeptidase (LAP), respectively. Further, alkaline phosphatase (AP) was used to test for phosphorus acquisition by adding 4-methylumbelliferyl-phospahte as a substrate. The microplates were incubated for 2 h at 15°C in the dark. Fluorescence was measured for hydrolytic enzyme activity (EA) using a Spectramax® Paradigm Multi-Mode Microplate Reader (Molecular Devices, USA). The hydrolytic enzymes PP and PO were evaluated using colorimetry and absorbance was measured on a Thermo Scientific Multiskan GO spectrophotometer (ThermoScientific, USA) (Sinsabaugh et al. 2008, German et al. 2011, De Scally et al. 2016).

### Statistical analysis

Statistical and exploratory data analyses were conducted using various packages in R version 4.2.2 (R Core Team 2010) and R studio desktop version: 2023.03.1+446 (Team 2020). Alpha and beta-diversity values were calculated from the rarefied dataset, using package “*phyloseq*” (v 1.38.0) (McMurdie and Holmes 2013)and “*microbiome R”* package (Leo and Shetty 2017). Significant differences were tested, using Wilcoxon rank-sum test, with *P*-value correction by FDR (Benjamini and Hochberg). Pairwise Permutational multivariate analysis of variance (PERMANOVA) was used to test for significant differences in microbial community abundance. The tests were conducted based on comparisons following nutrient addition between treatment groups, and the day of destructive sampling by using the *adonis* function at 999 random permutations with *P*-value correction (FDR) in R package “*vegan*” v.2.5.7 (Oksanen 2010). The differential abundances of microbiota, in response to nutrient amendment, were calculated with ANCOMBC-II (Lin and Peddada 2020) using the “*microeco”* package (v.0.19.0) in R (Liu et al. 2021a). Significant differences in soil pH, nutrient source (NS), microbial diversity (MD), and extracellular enzymatic activities between the treatments were tested and used to generate canonical correspondence analysis (CCA) ordination plots with the envfit function using “*microeco”* package (v 0.19.0). Soil parameters (pH, % of carbon, and % of nitrogen) and extracellular enzyme activities (LAP, AP, BX, BG, PO, and PP) were inspected for goodness of fit at a *P-value < .05 cut off. To establish the relationship between MD and extracellular EA, significantly differing microbial taxa were selected using RF (random forest + differential test) and correlation analysis (Karl Pearson) was performed for these differentially abundant taxa associated with soil microcosms at genus level and plotted with *P*-value significance with FDR correction all this was achieved again using the “*microeco*” package (v 0.19.0) (Liu et al. 2021a). The latent variable modeling was used to quantitatively evaluate the causal relationship between latent variables (MD, NS, and extracellular enzymatic activities) and their manifest variables via structural equation modeling using the *lavaan* package in R (Rosseel 2012). All plots were generated using the *ggplot2* (Wickham et al. 2016) and *ggpubr* v.0.4.0 (Kassambara 2020) supported with these packages in the RStudio environment.

## Results and discussion

### Nutrient augmentation affects chemical profile of soil microcosms

The pH was generally alkaline in most of the soil microcosm sets after nutrient and moisture amendment, with pH values as high as 13.73 (highly alkaline) in sample 4AZ and as low as 5.41 (acidic) in 1AW (Table S1). The alkaline pH in the control microcosm was consistent with coastal MDV soils (Aislabie et al. 1998). In treated microcosms, the addition of carbon and nitrogen substrates led to a decrease in soil pH (i.e. >1 unit decrease), while the addition of filter sterilized ultrapure water resulted in an increase in pH (i.e. 0.1–4 unit increase) when compared to the control (Table S1). Significant differences in pH were found between treatment groups of water with carbon, carbon and nitrogen with water and nitrogen with water (ANOVA, all ***P < .001). The soil pH may decrease due to ammonia oxidation or carbon dioxide release through microbial activity (Han et al. 2015, Ayiti and Babalola 2022). These decreases have also been shown to structure bacterial diversity and composition (Li et al. 2011). In general, high diversity is prevalent in neutral soils and lower diversity is typically found in acidic or alkaline soils (Zhalnina et al. 2015). Nitrogen levels were lowest (0.03%) among untreated Antarctic soils (control), and highest (1.23%) in carbon and nitrogen supplemented soils. As expected, the levels of nitrogen detected were highest (0.51%) in nitrogen source supplemented soils, similarly carbon levels were higher in all soils supplemented with carbon source, with a maximum of 2.48% in 1CW. The carbon and nitrogen concentrations increased over time, in treated soils, with glucose and glycine treated soils showing the highest percentages of carbon (0.36%–2.48%) and nitrogen (0.21%–1.23%), respectively (Table S1). These observations confirm the validity of nutrient amendments as the increased carbon and nitrogen values were directly proportional to the supplied treatments in comparison to the unamended control.

### MD changes disproportionately in response to carbon, nitrogen, and moisture addition

**Figure 1.**
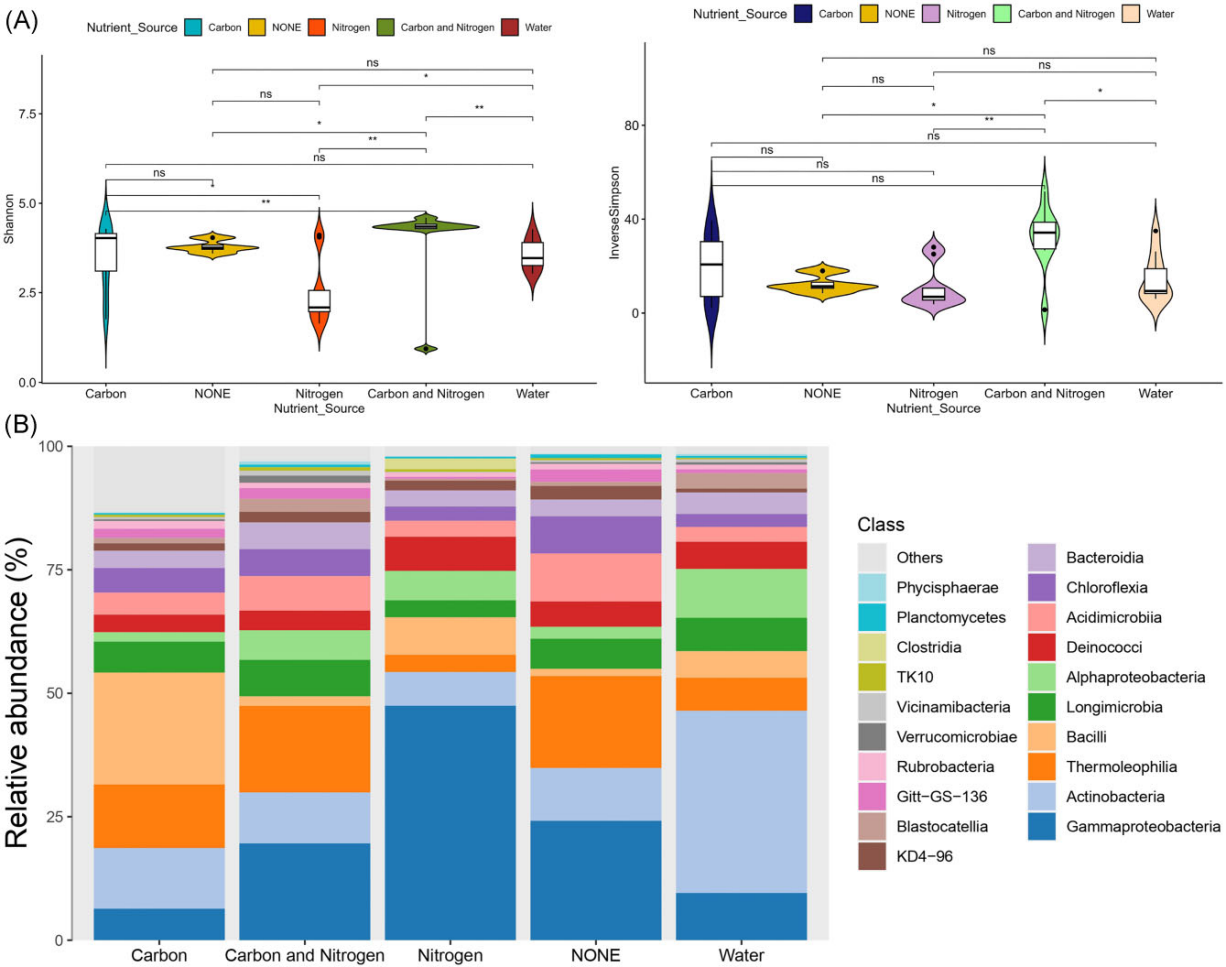
(A) Violin plots depict the Shannon and inverse Simpson diversity of the Antarctic soil microcosms upon moisture and nutrient supplementation. The significance was tested using Wilcoxon rank-sum test with *P*-value correction using FDR (Benjamini and Hochberg). Significant differences are marked by asterix (**P < .01; *P < .05), ns stands for nonsignificant. (B) Relative abundance of top 20 bacterial class in Antarctic soil microcosms upon moisture and nutrient supplementation. “Others” represent the proportion of less abundant class.

**Figure 2.**
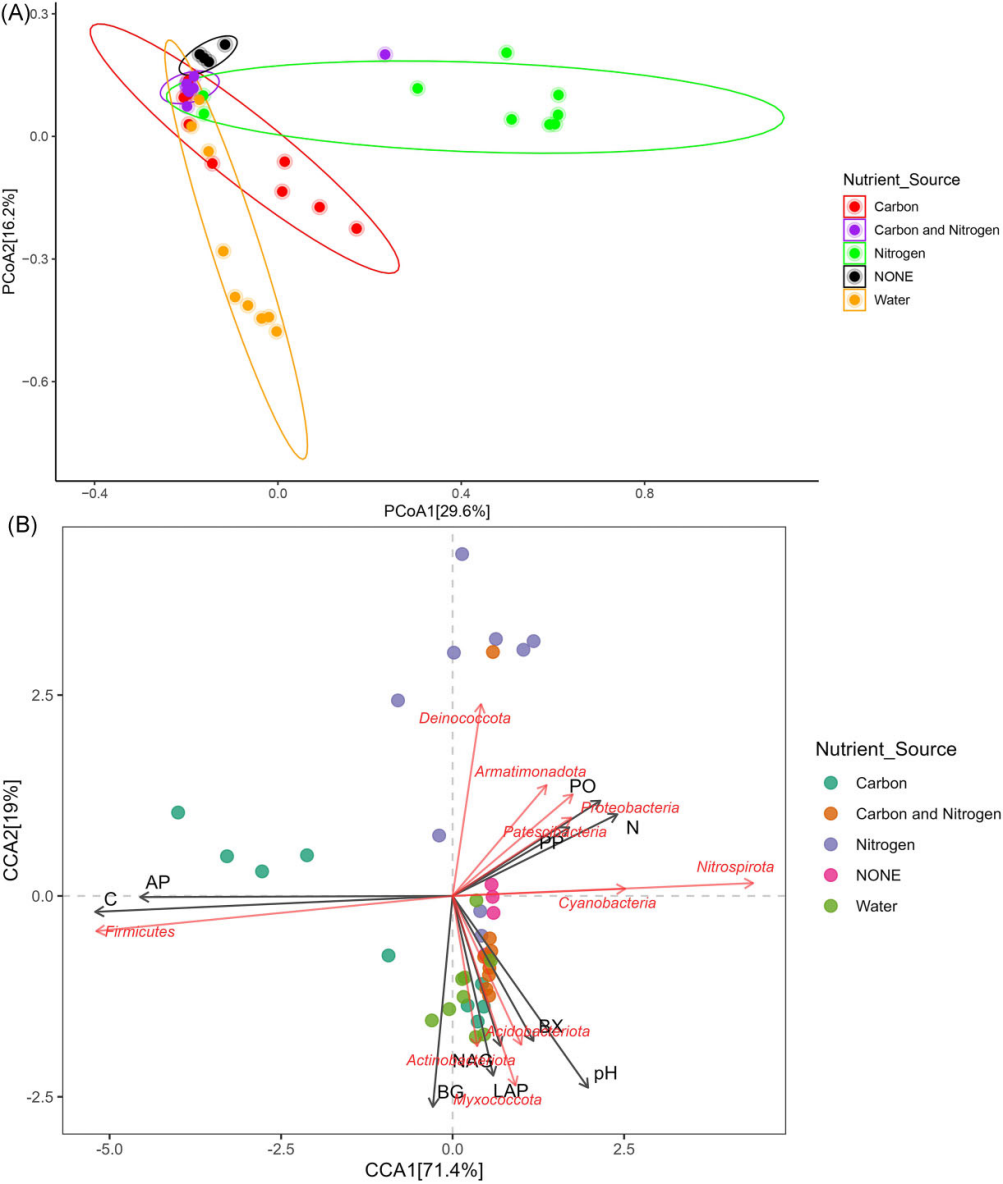
(A) PCoA plot (Bray–Curtis distance) showing abundance and distribution of ASVs upon nutrient and moisture amendment (Wilcox, ***P < .01; *P < .05). (B) CCA plot based on Bray–Curtis distance showing the effect of extracellular enzymatic activity and soil parameters on Antarctic soil microbial communities under different treatment regimes.

The addition of moisture, carbon, nitrogen, and carbon with nitrogen containing substrates to soils resulted in a significant difference (Wilcox, *P < .05; **P < .01) in the diversity of microbiota among the treatment sets (Fig. 1A). Alpha-diversity measurements (Shannon and inverse Simpson index) were high in soils supplemented with carbon and nitrogen combined, as opposed to nitrogen addition where a substantial reduction in MD was observed (Table S2). This shows that the combined addition of carbon and nitrogen favors the Antarctic Dry Valley soil microbial communities in contrast to addition of nitrogen with decreased effects in a controlled environmental setup (microcosm). Earlier report on the effects of glycine (carbon and nitrogen combined) addition on Antarctic soil microbial communities has shown varied responses in two different sampling sites wherein glycine addition led to increase in Gram positive bacteria indicated by high concentrations of ester-linked fatty acids (ELFAs) in one sampling site compared to the control (Dennis et al. 2013a). Following this using ELFAs, another study carried out specifically on Antarctic Dry Valley soil microbial communities showed that high nitrogen amendment reduced the total ELFA concentration. Contrastingly, the ELFA-linked Shannon and Simpson diversity were reported to decrease only with high carbon and high carbon combined with low nitrogen treatments compared to the other treatment sets (Dennis et al. 2013b). While a previous study before this carried out in Antarctic Dry Valley soil has reported no evident changes in microbial community structure after nutrient supplementation and concluded that the microbial community is unresponsive to treatment (Hopkins et al. 2008). A newer study on Antarctic soil microbial communities using 16S rRNA gene sequencing also reported no direct effect of nutrient application supplied in the form of tryptic soy broth on bacterial community composition or diversity (Newsham et al. 2019). Considering these discrepancies from the earlier observations on the response of Antarctic soil microbial communities to nutrient treatments, we presume that nutrient treatments induce considerable shifts in the microbial community structure and stability of the microbial community depends on several other factors influencing the nutrient availability and its uptake in the Antarctic soil ecosystem. Arguably, as opposed to these earlier studies that are carried out in the field (*in situ*) ours is a closed system (*ex situ*) wherein microorganisms are neither added nor removed and maintained in a controlled manner. Hence, we predict that these reductions or improvements may be because of specific responses of some microbial taxa to nutrient input which needs further understanding. Principal coordinate analysis (PCoA), based on Bray–Curtis dissimilarity matrix, suggests a significant variation (Wilcox, ***P < .01; *P < .05) among the microbial communities following nutrient-addition. The microbial communities treated with moisture, carbon, and carbon along with nitrogen were broadly similar compared to the nitrogen treatment (Fig. 2A). Further a significant difference in the community composition as a response to nutrient and moisture addition was also tested using pairwise permanova that showed significance (PERMANOVA, *P < .05; **P < .01) among all tested pairs except for none (control) vs carbon and nitrogen (Table S3). We did not find significant differences based on samples collected at different time points (days). These findings suggest that nutrient amendments may disproportionately affect Antarctic soil microbial communities.

### Addition of nutrients may favour copiotrophic microbial communities over oligotrophic

The analysis of ASV relative abundance showed that ASVs belonging to bacterial taxa were the most dominant in Antarctic soil microcosms (99%) compared to archaea, which constituted minor fractions of these ASVs (Fig. S2b). The results are consistent with previous reports (Makhalanyane et al. 2013, Lambrechts et al. 2019, Barnard et al. 2020, Malcheva et al. 2020, Ortiz et al. 2021) that show polyextreme soils harbor surprisingly few archaea, which might be a reason to observe this result in our dataset apart from several others. *Crenarchaeota*, now *Thermoproteota*, were the only prominent archaea recovered in our samples. Members of the phylum *Thermoproteota* are thermostable anaerobic Archaea, typically found in the Antarctic soils (Hatzenpichler et al. 2008, Lewis et al. 2021, Kochetkova et al. 2022). Archaea from this acidophilic phylum require elemental sulphur (S°) for respiration, although their capacity to use carbon and nitrogen sources remains unclear (Florentino et al. 2016). These archaea (*Nitrososphaeria*) decreased significantly (*P < .05, ANCOMBC-II) in relative abundance following moisture addition (Fig. S3a), compared to control. These archaeal lineages might be better adapted to survive in low moisture conditions, as the soil was collected from Antarctic Dry Valley, which receives very low precipitation (Vishnivetskaya et al. 2018, Greenfield et al. 2020) .

Bacterial ASVs associated with Antarctic soil microcosms, were broadly affiliated with 20 dominant class including *Gammaproteobacteria, Actinobacteria, Thermoliophilia, Bacilli, Longimicrobia, Alphaproteobacteria, Deinococci, Acidimicrobiia, Chloroflexia, Bacteroidia*, and several others (Fig. 1B). Our analysis confirmed that nutrient augmentation resulted in significant changes (***P < .001, ANCOMBC-II) in the relative abundances of these dominant members of bacterial class (Table 1). *Proteobacteria* (*Gammaproteobacteria*) appeared to respond positively to nitrogen input compared to other treatments (Table 1) and poorly to carbon input among the carbon and control group as confirmed by the significant decrease in their relative abundance pattern (Fig. S3b). Further, addition of moisture led to decrease in relative abundance of *Actinobacteriota* (*Thermoliophilia* and *Acidimicrobiia*), *Chloroflexi* (*Chloroflexia, KD4-96*, and *Gitt-GS-136*), *Armatimonadota* (*Armatimonadia*), *and Patescibacteria|* (*Saccharimonadia*) in moisture vs control microcosm (Fig. S3a). Nitrogen addition also favoured *Acidobacteriota* (*Acidobacteriae*) when compared to control microcosm (Fig. S3c), and *Firmicutes* (*Bacilli* and *Clostridia*) but negatively impacted *Verrucomicrobiota* (*Chlamydiae* and *Verrucomicrobiae*) in all treatment comparisons (Table 1). We also found significant increases in the abundance of *Acidobacteriota* (*Holophagae, Blastocatellia*, and *Acidobacteriae*), *Bacteroidota* (*Kapabacteria*) and *Gemmatimonadota* (*S0134 terrestrial group*) after combined addition of carbon and nitrogen in comparison to control (Table 1, Fig. S3d). Nonmetric multidimensional scaling (NMDS) ordination analyses, based on Bray–Curtis distances of bacterial community data (Fig. S2a), showed that the different nutrient treatments resulted in significant (***P < .001, PERMANOVA) structural differences among microcosm communities. We observed differences in the structural patterns of bacterial communities following the sole addition of nitrogen. Compared to other treatments which resulted in random distribution patterns, nitrogen addition appears to be the only treatment leading to clear community structural patterns (Fig. S2) and having contrasting effects on the relative abundances of *Proteobacteria* and *Verrucomicrobiota* (Table 1). *Proteobacteria* typically grow and reproduce rapidly in high nitrogen environments, due to their eutrophic physiology (Fierer et al. 2012, Ma et al. 2021), which may explain their positive responses to nitrogen addition. The significant increase in *Actinobacteriota* (*Thermoliophilia* and *Acidimicrobiia*) abundance (Fig. S3a) with respect to moisture addition, may be explained by their well-known capacity to rapidly respond to precipitation (Koyama et al. 2018). Based on these findings, it appears that nutrient augmentation leads to significant shifts in bacterial community composition, which are random and not specific to members of bacterial class. However, sole effects of nitrogen treatment suggest that copiotrophic lineages might be more supported, in contrast to oligotrophic lineages, which are found ubiquitous in these Antarctic soils (Fierer et al. 2007, Koyama et al. 2014, Ho et al. 2017, Ma et al. 2021).

**Table 1.** Differentially abundant bacterial taxa in Antarctic soil microcosms with response to nutrient and moisture amendment.

Comparison	Taxa	*P*.adj	Sig	Group
Carbon—NONE	*Proteobacteria (Gammaproteobacteria)*	0.000615	***	NONE
Carbon—Nitrogen	*Proteobacteria (Gammaproteobacteria)*	9.54E–06	***	Nitrogen
Carbon—Nitrogen	*Patescibacteria (Parcubacteria)*	7.34E–06	***	Nitrogen
Carbon—Nitrogen	*Acidobacteriota (Holophagae)*	0.000672	***	Carbon
Carbon—Nitrogen	*Patescibacteria (Saccharimonadia)*	0.000715	***	Carbon
Carbon—Nitrogen	*Chloroflexi (P2-11E)*	1.30E–05	***	Carbon
Carbon—Nitrogen	*Bdellovibrionota (Oligoflexia)*	8.62E–05	***	Carbon
Carbon—Nitrogen	*Verrucomicrobiota (Chlamydiae)*	2.23E–07	***	Carbon
Carbon—Nitrogen	*Bdellovibrionota (Bdellovibrionia)*	1.25E–09	***	Carbon
Carbon—Water	*Chloroflexi (Gitt-GS-136)*	3.82E–05	***	Carbon
NONE—carbon and nitrogen	*Acidobacteriota (Blastocatellia)*	2.74E–05	***	Carbon and nitrogen
NONE—carbon and nitrogen	*Bacteroidota (Kapabacteria)*	2.59E–06	***	Carbon and nitrogen
NONE—carbon and nitrogen	*Acidobacteriota (Holophagae)*	0.000313	***	Carbon and nitrogen
NONE—water	*Chloroflexi (Chloroflexia)*	5.97E–05	***	NONE
NONE—water	*Chloroflexi (KD4-96)*	5.55E–05	***	NONE
NONE—water	*Chloroflexi (Gitt-GS-136)*	0.000197	***	NONE
NONE—water	*Gemmatimonadota (Gemmatimonadetes)*	8.44E–05	***	Water
NONE—water	*Patescibacteria (Saccharimonadia)*	0.000278	***	NONE
Nitrogen—carbon and nitrogen	*Firmicutes (Bacilli)*	0.000168	***	Nitrogen
Nitrogen—carbon and nitrogen	*Firmicutes (Clostridia)*	7.34E–06	***	Nitrogen
Nitrogen—carbon and nitrogen	*Verrucomicrobiota (Chlamydiae)*	1.67E–06	***	Carbon and nitrogen
Nitrogen—carbon and nitrogen	*Bdellovibrionota (Oligoflexia)*	7.22E–08	***	Carbon and nitrogen
Nitrogen—carbon and nitrogen	*Patescibacteria (Parcubacteria)*	2.27E–12	***	Nitrogen
Nitrogen—water	*Proteobacteria (Gammaproteobacteria)*	1.17E–08	***	Nitrogen
Nitrogen—water	*Verrucomicrobiota (Verrucomicrobiae)*	0.000129	***	Water
Nitrogen—water	*Bacteroidota (Rhodothermia)*	0.000786	***	Water
Nitrogen—water	*Sumerlaeota (Sumerlaeia)*	8.28E–09	***	Water
Nitrogen—water	*Bacteroidota (Kapabacteria)*	2.68E–15	***	Water
Carbon and nitrogen—water	*Actinobacteriota (Actinobacteria)*	3.63E–05	***	Water
Carbon and nitrogen—water	*Firmicutes (Bacilli)*	1.28E–06	***	Water

Column “Group” contains the treatment category, which has increased abundance for the respective microbial phyla and class, *P*-values adjusted through FDR using ANCOMBC-II. “Sig” denotes significance, and “NONE” denotes no nutrient amendment.

### Antarctic soil microbial communities show positive and negative correlations with soil parameters and extracellular enzymes under different treatment regimes

**Figure 3.**
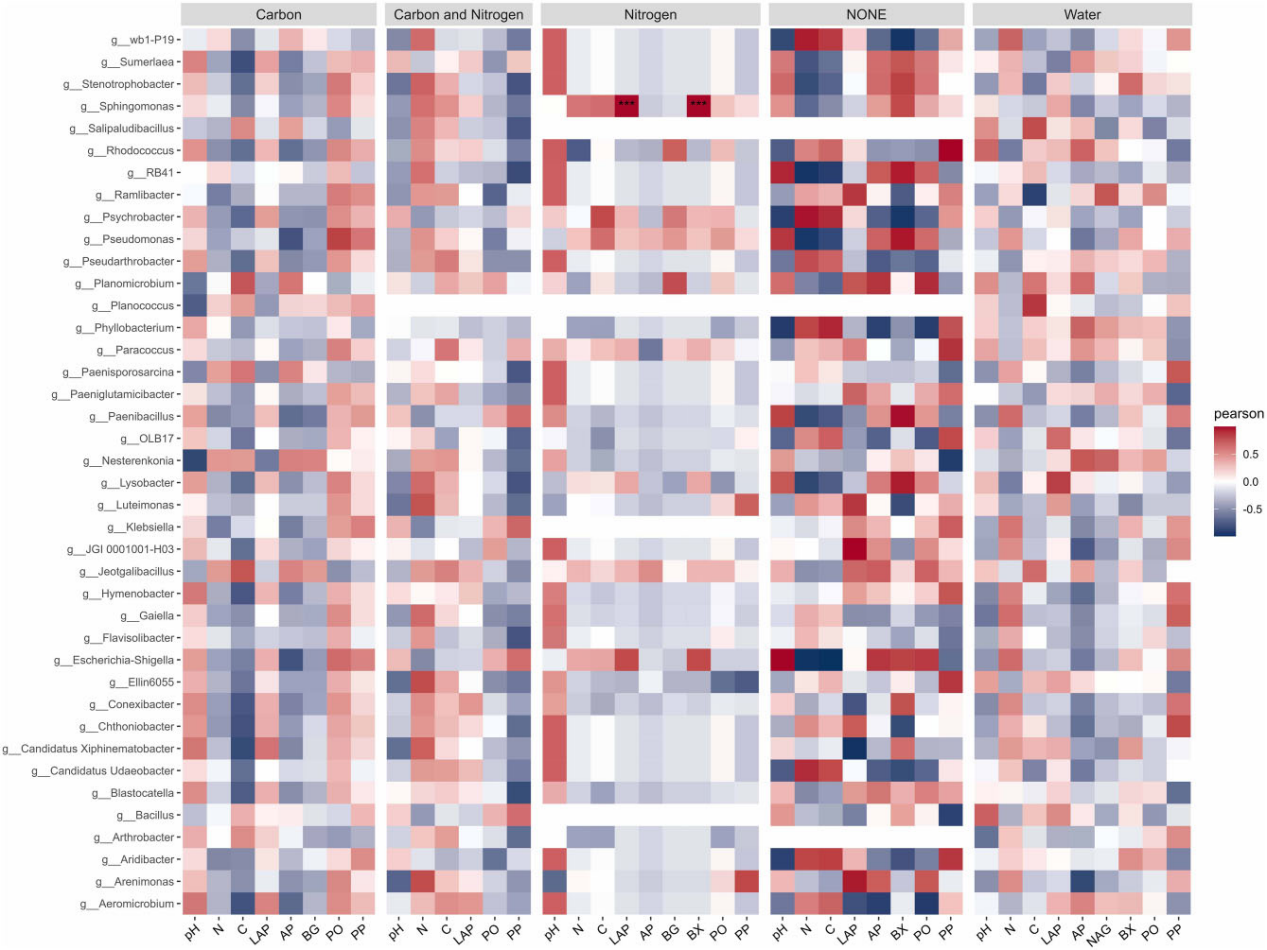
Correlation plot showing the correlation between extracellular EA, soil parameters, and microbial genera for different treatment sets in the Antarctic soil microcosm. The significance of Pearson correlation is marked by asterisks in the plot (***P < .001). g_: denotes genus.

The extracellular enzyme activities showed different values under different treatment regimens with no distinct pattern (Fig. S4). The LAP activity declined after addition of carbon compared to other treatments (Fig. S4a), which was completely opposite to EA of AP (Fig. S4b). The NAG activity was seen higher with moisture, nitrogen, and carbon and nitrogen input in relation to control and carbon (Fig. S4c). The activity of PO (Fig. S4f) and PP (Fig. S4g) were observed only high with carbon and control sets, whereas it was low in other treatment sets. Addition of nitrogen and moisture positively influenced the activity of BG when compared to carbon and carbon and nitrogen together (Fig. S4d), while in BX all treatments had positive effect with respect to control (Fig. S4e). Further, the relationship between extracellular enzymatic activities and soil parameters on microbial community structure in the different soil microcosms, was studied. We used constrained ordination, through CCA, to assess the factors (soil parameters and extracellular enzymatic activities) and test their correlations using relative abundance of microbial communities in different microcosms. Only those factors that showed strong significant correlations (*P < .05) were used to visualize the CCA by plotting graph. Overall, Antarctic soil microbial community abundances were positively influenced by several factors including pH, nitrogen availability and metabolic activity of extracellular enzymes such as LAP, AP, BX, PO, and PP. In contrast, we found that these soil communities were negatively impacted by carbon input and activity of extracellular enzyme BG (Fig. 2B). Correlation coefficient analysis (Pearson), conducted at genus level on individual treatment sets, further corroborated the extent to which soil parameters and extracellular enzymatic activities influenced microbial abundances (Fig. 3). The correlations were only significant for soils amended with nitrogen. Within the group amended with nitrogen, extracellular enzymatic activities of LAP and BX were significantly correlated (***P < .001) with members of the genus *Sphingomonas*. Positive and negative correlations, observed with other microcosm treatment sets supplemented with nitrogen, carbon, both nitrogen and carbon combined, water were not significant (Fig. 3). Extracellular enzymatic activities are key indicators of microbial function and provide some reflection on microbial contributions to nutrient cycling. While some recent studies have criticized the use of these enzymes (Mori et al. 2023), there is strong evidence that these enzymes provide valid data for determining the shifts in microbial communities (Xiao et al. 2018, Yang et al. 2020, Gao et al. 2021, Ma et al. 2021). Assessing extracellular enzymatic activities may provide some indication of the efficiency of nutrient utilization in these oligotrophic environments (Robertson 1999, Rovira and Vallejo 2002, Veres et al. 2015, Huang et al. 2020). The positive correlation (***P < .001) found for LAP and genus *Sphingomonas* suggests a direct link between nitrogen use efficiency in soils supplemented with nitrogen (Fig. 3). Our analyses suggest that several other factors had correlations (both positive and negative) with nutrient and moisture supplementation in Antarctic soil microcosms. However, these correlations were not significant enough to explain nutrient utilization capacity in these Antarctic soil microcosms. In summary, it appears that only bacterial genus *Sphingomonas* present in the Antarctic soil microbial community had the capacity to use the nutrient substrates and demonstrated significant nutrient utilization efficiency that requires further research for fully understanding the mechanism and implications, while changes in the abundance pattern *Sphingomonas* have been shown to have a direct link with nitrogen addition in agricultural soils (Galindo et al. 2021). They are also known to promote plant growth by their ability of fix atmosphere nitrogen and improve nitrogen supply indicating their nitrogen use efficiency (Luo et al. 2019, Zhang et al. 2023). The analysis revealed the effects of extracellular enzymatic activities and soil parameters to changes in microbial taxa, following nutrient addition and the results showed significance impacts of nitrogen addition in these oligotrophic soils.

### Structural equation modeling revealed significant relationship among NS, extracellular enzymatic activity, and MD

**Figure 4.**
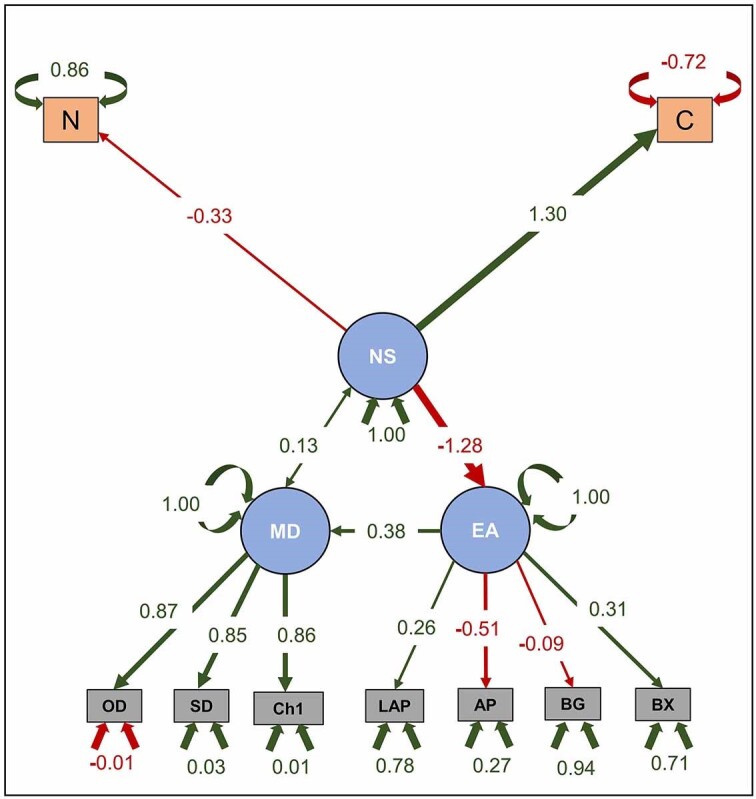
Structural equation model depicting the relationship among EA, NS, and MD. Width of the arrow path corresponds to the significance level of associations. OD: observed diversity; SD: Shannon diversity; Ch1: Chao1; N: nitrogen; C: carbon; LAP: leucyl aminopeptidase; AP: Alkaline phosphatse; NAG: ß-*N*-acetylglucosaminidase; BG: ß-1,4-glucosidase; BX: ß-1,4-xylosidase; PO: phenol oxidase; and PP: phenol peroxidase.

Structural equation modeling (SEM) was used to predict the causal relationships among microbial communities and their primary drivers in Antarctic soil microcosms, in response to nutrient addition (Mamet et al. 2019). SEM predicted significant relationships among NS, extracellular EA , and MD. Both EA and NS were positive predictors of MD in Antarctic soil microcosms with enzymatic activities having a stronger effect (0.38) compared to NS (0.13), However the effect of the NS, on EA, was relatively weak (−1.28). SEM suggests that the measured environmental parameters had a positive effect on the latent variables except for observed diversity, nitrogen source, BG, and AP, which had a negative relationship with the predictive components (Fig. 4). Extracellular enzymatic activities, ascribed chiefly to LAP and BG, were a positive predictor of MD in Antarctic soil microcosms. This is consistent with previous reports which showed positive relationships between soil extracellular enzymatic activities and MD (Van Horn et al. 2014, Geyer and Barrett 2019) In contrast to the effects of other extracellular enzymes including BG, BX, AP, NAG, PO, and PP, LAP activity provides insights regarding the acquisition of nitrogen in these Antarctic soils (Sinsabaugh et al. 2008, Bragazza et al. 2019). NS, marked by nitrogen and carbon, positively predicted MD. However, the relationship between NS and EA was less strong (Fig. 4). The extent to which nitrogen addition drives MD in soil remains unclear. This is because we could not corroborate the relationship between microbial communities and nitrogen addition due to the degree of variability (Williams et al. 2013, Zhao et al. 2014). However, there are some insights from comparable systems suggesting a strong relationship between nitrogen supplementation and microbial communities. For instance, a recent study reported that nitrogen addition substantially altered MD, with significant increases in the abundances of soil bacteria and archaea in permafrost peatlands (Ma et al. 2021). A separate study also used SEM analysis to demonstrate a negative influence of nutrient addition on enzymatic activities showing that LAP was directly affected by nitrogen addition (Schnecker et al. 2014).

## Conclusion

There is strong evidence that the melting of buried, and surface ice has led to the mobilization of soil nutrients due to run-off. However, there effects of nutrient mobilization on microbial diversity and functionality remains unclear. Given the centrality of microbial communities as drivers of Antarctic food webs, nutrient mobilization may substantially influence ecosystem services. In this study, we used an experimental manipulation to investigate the effect of changes in Antarctic soil microorganisms and their potential functionality by adding nutrient and moisture supplements in a microcosm. The addition of nitrogen, carbon, both carbon and nitrogen and water, over a 45-day period, had pronounced effects on microbial diversity. The results from our studies suggest that increases in the availability of nitrogen and carbon may result in substantial changes in microbial community structure and diversity. The altered nutrient regimes, due to the mobilization of nutrients, may result in significant changes in dominant bacterial taxa, including *Proteobacteria* (*Gammaproteobacteria*), *Firmicutes* (*Bacilli* and *Clostridia*), *Actinobacteriota* (*Thermoliophilia* and *Acidimicrobiia*), *Chloroflexi* (*Chloroflexia, KD4-96*, and *Gitt-GS-136*), *Acidobacteriota* (*Holophagae, Blastocatellia*, and *Acidobacteriae*), and *Verrucomicrobiota* (*Chlamydiae* and *Verrucomicrobiae*). The results from SEM analysis suggest a positive correlation between nutrient addition and extracellular enzymatic activities on the diversity of Antarctic soil microbial community. Taken together, the significant changes in microbial diversity and the related extracellular enzymatic activities indicate that certain taxa may respond more rapidly to shifts in nutrient regimes in climate-sensitive region of Antarctica. This finding is in contrast with earlier studies that report Antarctic microorganisms may be resistant to such changes (Hopkins et al. 2008, Sparrow et al. 2011, Dennis et al. 2013a, Newsham et al. 2019). Significant shifts in the composition and function of Antarctic soil microbes suggest that these communities may be resilient, and not resistant to ecosystem changes which is critical to understanding ecosystem stability with respect to climate driven changes. Future studies may however provide insights regarding the implications of this microbial resilience on ecosystems services, and Antarctic food webs.

## Supplementary Material

fiae071_Supplemental_Files
