# Supplementary figures and images for: Changes in nutrient availability substantially alter bacteria and extracellular enzymatic activities in Antarctic soils
Source: FEMS Microbiol Ecol. 2024 May 2;100(6):fiae071. doi: 10.1093/femsec/fiae071 (PMC11107947; doi:10.1093/femsec/fiae071)

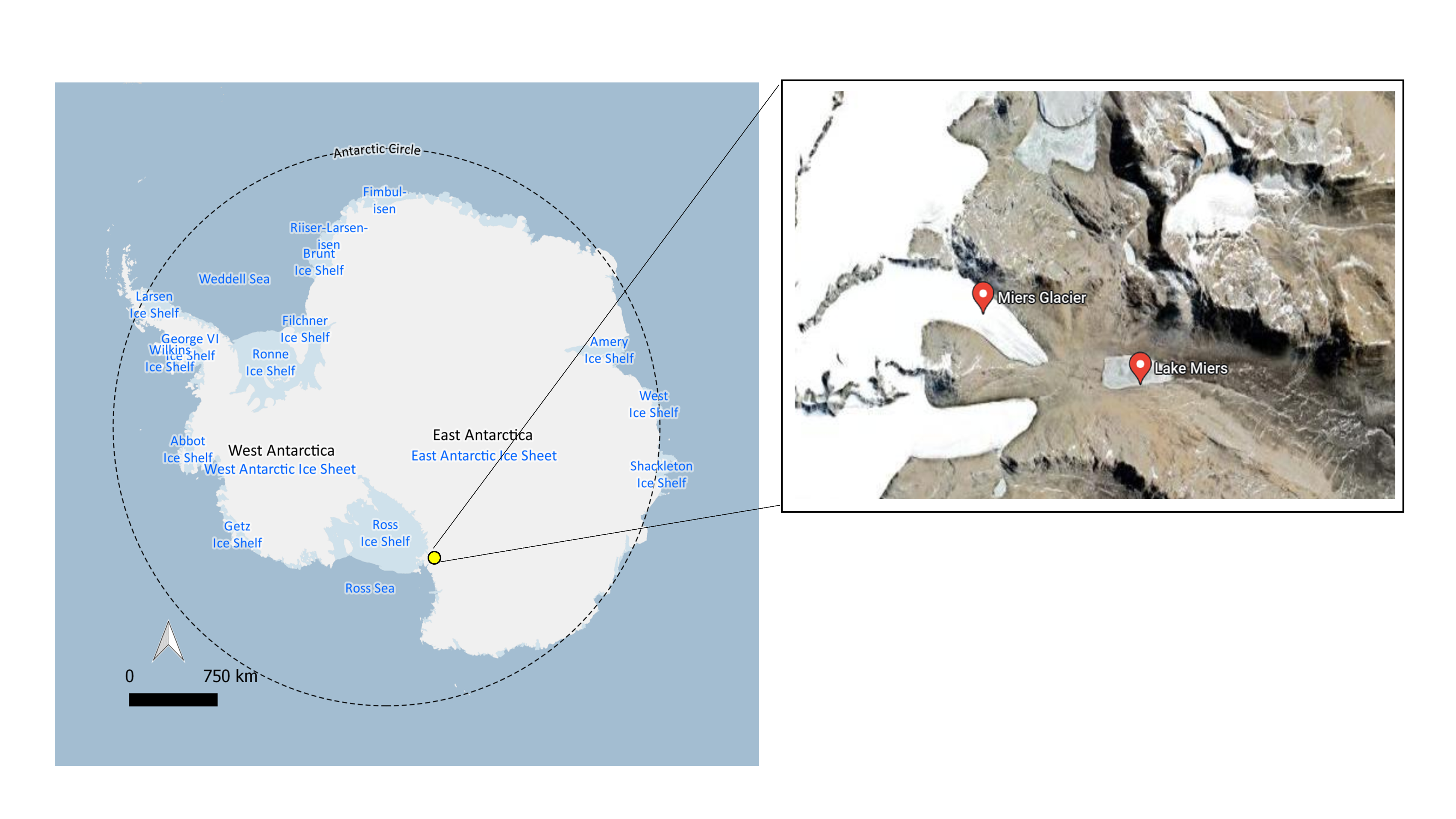

Supplement: fiae071_Supplemental_Files [file fiae071_supplemental_files.zip › supp data Fig.S1.tif]

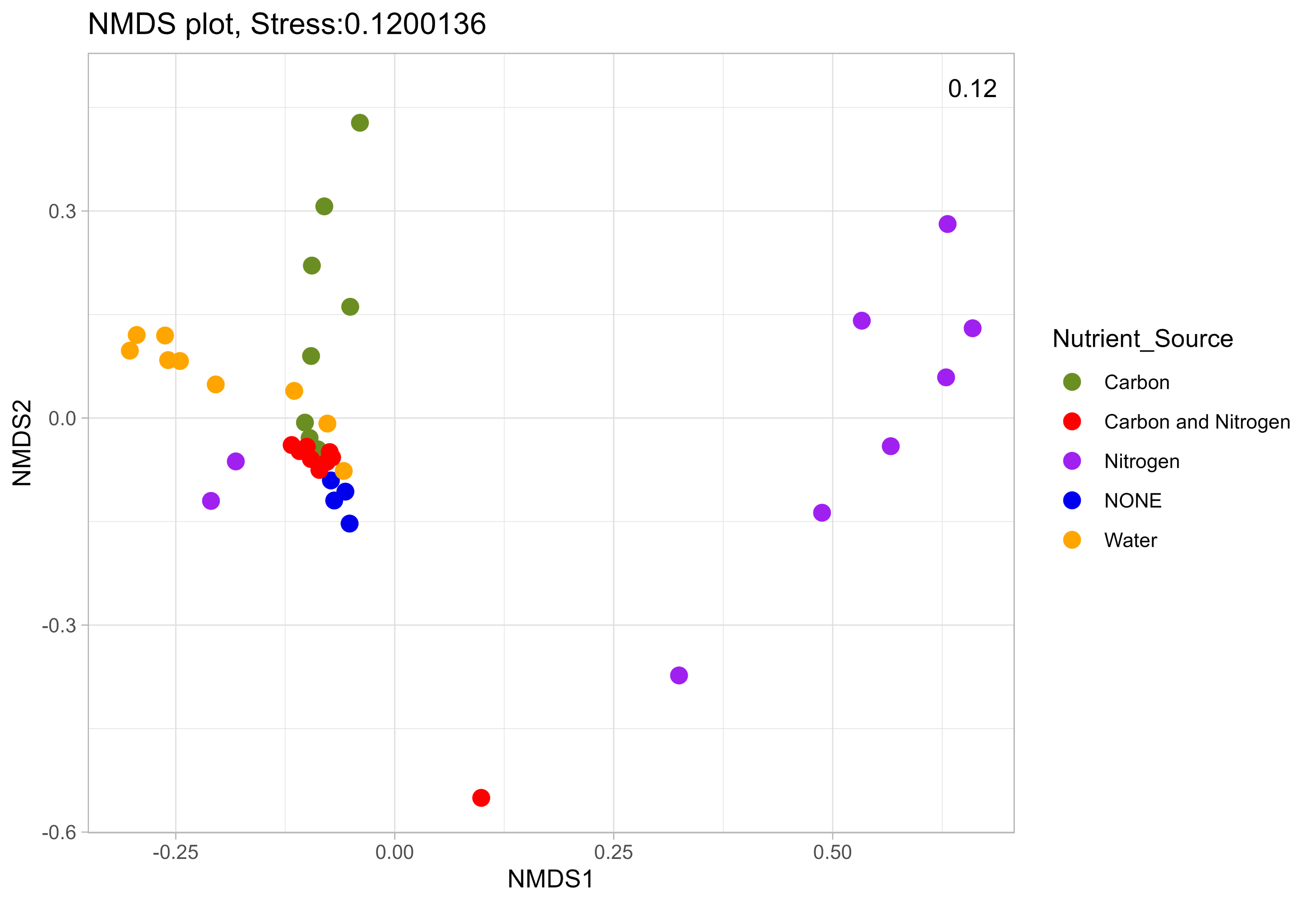

Supplement: fiae071_Supplemental_Files [file fiae071_supplemental_files.zip › supp data Fig.S2a.tif]

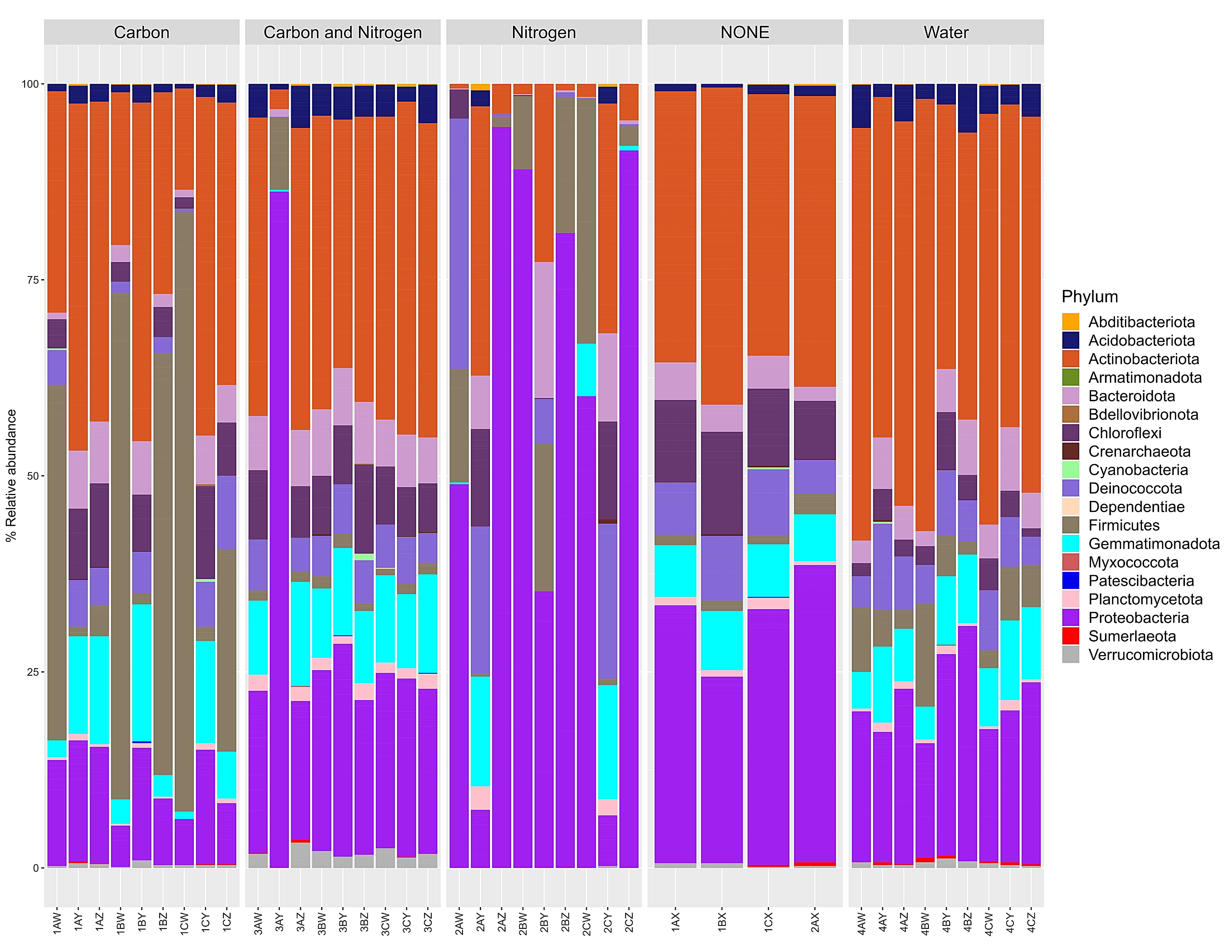

Supplement: fiae071_Supplemental_Files [file fiae071_supplemental_files.zip › supp data Fig.S2b.tif]

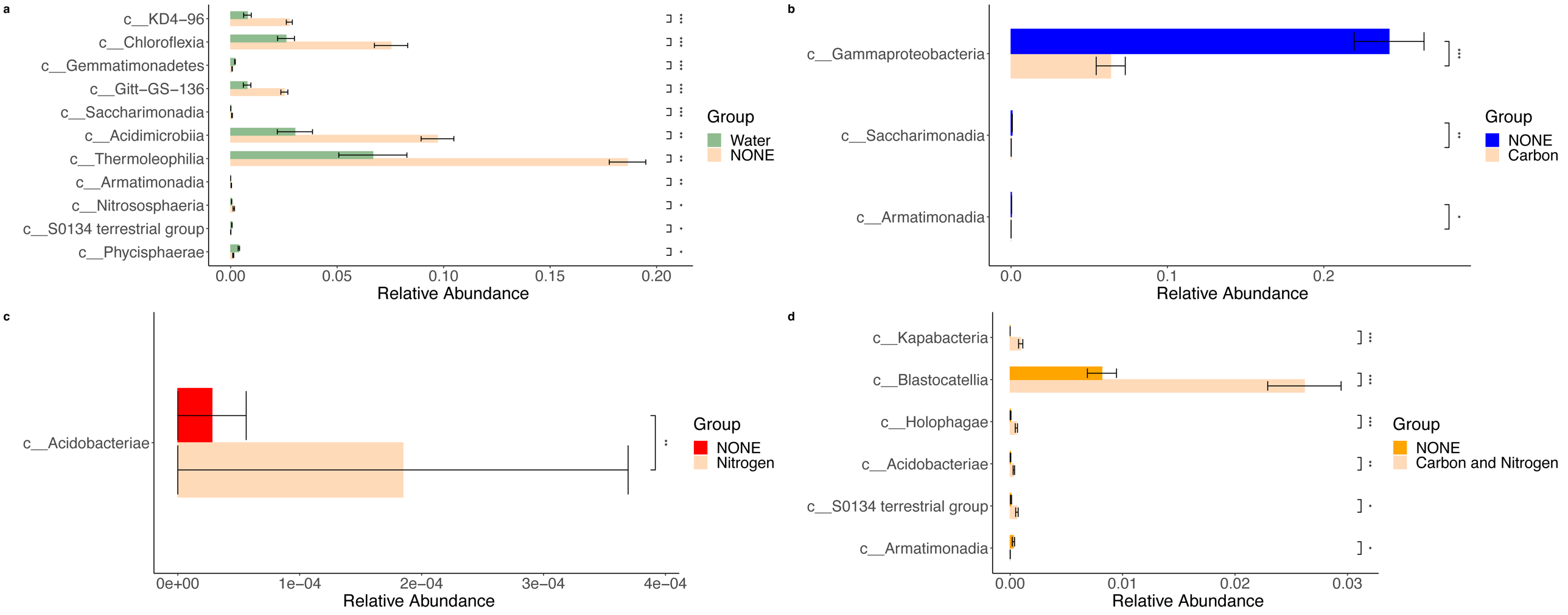

Supplement: fiae071_Supplemental_Files [file fiae071_supplemental_files.zip › supp data Fig.S3.tif]

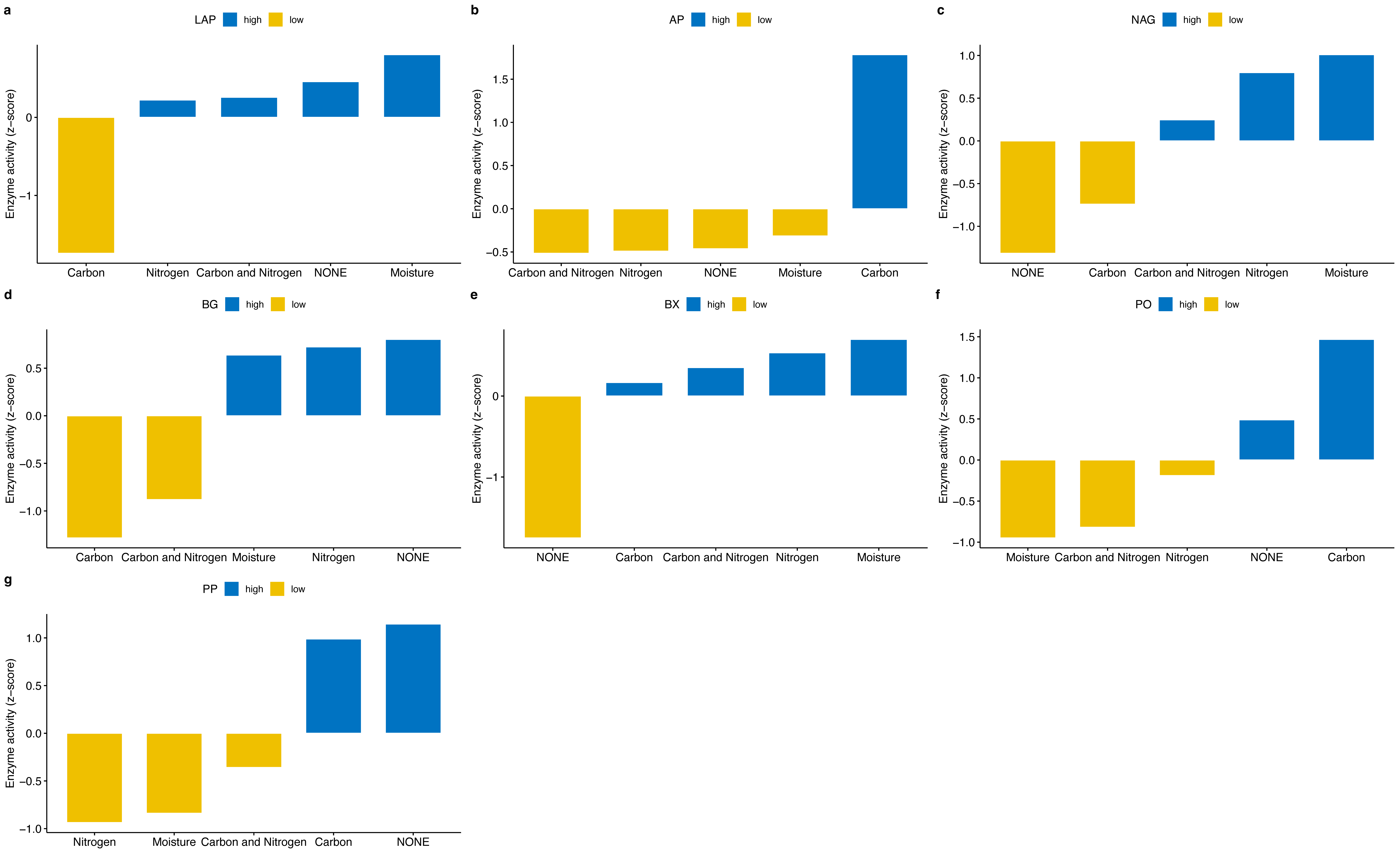

Supplement: fiae071_Supplemental_Files [file fiae071_supplemental_files.zip › supp data Fig.S4.tif]

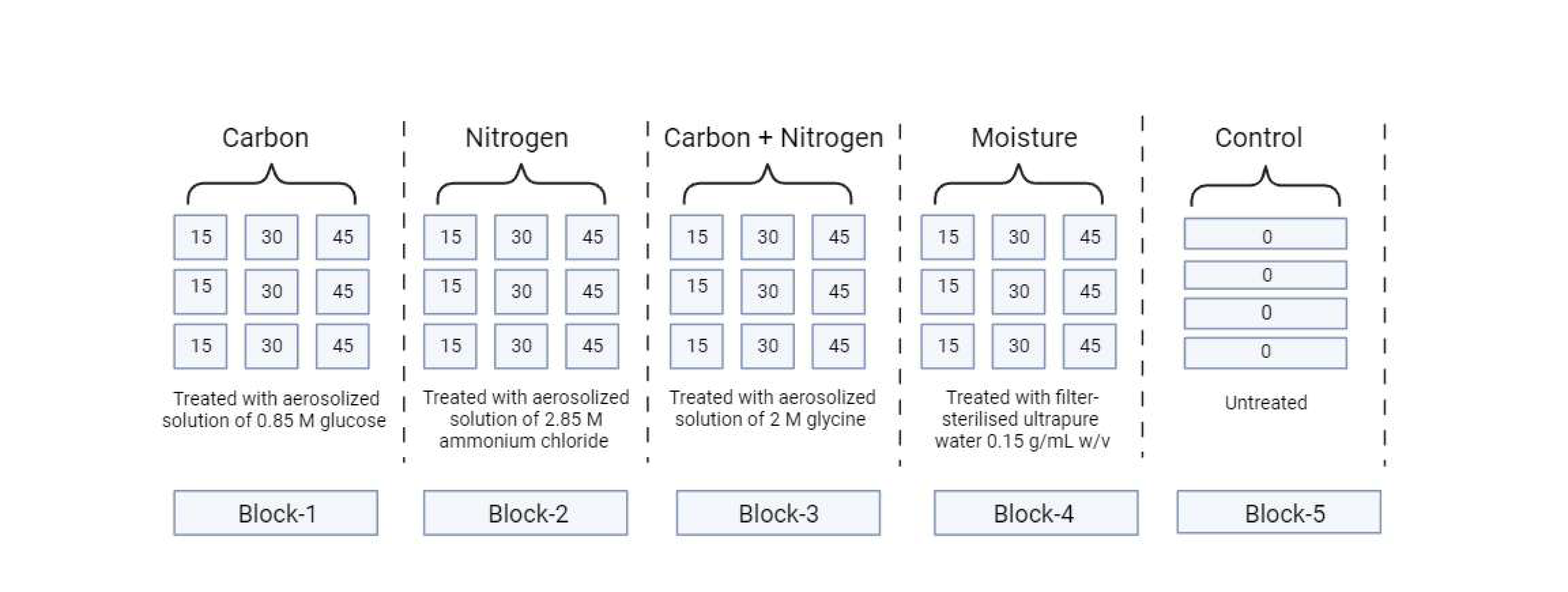

Supplement: fiae071_Supplemental_Files [file fiae071_supplemental_files.zip › supp data Fig.S5.tif]
